# Macroscopic Evaluation of Poly(3-hydroxybutyrate-co-3-hydroxy valerate), PHBV-Based Nanofiber Scaffolds with Aloe Vera or Honey in Murine Wound Healing

**DOI:** 10.3390/pharmaceutics17070833

**Published:** 2025-06-26

**Authors:** José Manuel Pérez-Galván, José Enrique Hernández-Rodríguez, José Luis Martín-Barrasa, Maximina Monzón-Mayor, Pedro Saavedra-Santana, María del Mar Romero-Alemán

**Affiliations:** 1 Advanced Confocal and Electron Microscopy Research Facility (SIMACE), University of Las Palmas de Gran Canaria, 35016 Las Palmas de Gran Canaria, Spain; jose.perezgalvan@ulpgc.es; 2 University Institute for Biomedical and Health Research, Department of Nursing, University of Las Palmas de Gran Canaria, 35016 Las Palmas de Gran Canaria, Spain; 3 Research Unit, Animal Facility, Hospital Universitario de Gran Canaria Dr. Negrín, Barranco de La Ballena s/n, 35019 Las Palmas de Gran Canaria, Spain; joseluis.martin@ulpgc.es; 4 IUSA-ONE HEALTH 2—Sanidad Animal de la Acuicultura y Especies Silvestres, Enfermedades Infecciosas y Seguridad Alimentaria, University Institute of Animal Health and Food Safety (IUSA), University of Las Palmas de Gran Canaria, 35416 Arucas, Spain; 5 Eukaryotic-Prokaryotic Synergy—Comparative and Translational Medicine (Cardiorespiratory Infectious Diseases and Epidemiology Group), Fundación Canaria del Instituto de Investigación Sanitaria de Canarias (FIISC), 35012 Las Palmas de Gran Canaria, Spain; 6 CIBER de Enfermedades Infecciosas (CIBERINFEC), Instituto de Salud Carlos III, 28029 Madrid, Spain; 7 University Institute for Biomedical and Health Research, Department of Morphology, University of Las Palmas de Gran Canaria, 35016 Las Palmas de Gran Canaria, Spain; maximina.monzonmayor@ulpgc.es; 8 Department of Mathematics, University of Las Palmas de Gran Canaria, 35016 Las Palmas de Gran Canaria, Spain; pedro.saavedra@ulpgc.es (P.S.-S.);

**Keywords:** nanopolymers, scaffolds, PHBV, aloe vera, honey bee, electrospinning, tissue engineering, wound healing

## Abstract

**Background/Objectives**: The utility of various biocompatible biological and synthetic polymers as substrates to provide structural support, facilitate cell migration, and promote the healing of full-thickness wounds by secondary intention has been studied. This includes intelligent structures that enable the release of natural products or drugs for these and other purposes. In this study, the primary objective was to analyze and compare, from a macroscopic perspective, the individual behavior of the polymer poly (3-hydroxybutyrate-co-3-hydroxyvalerate) (PHBV), with Aloe vera (PHBV/Av) or honey (PHBV/Ho), in the healing process of a full-thickness skin wound over 40 days in a murine model, in addition to describing the microscopic ultrastructure of the nanofibers. **Methods**: Two experimental groups were established, PHVB/AV (*n* = 5) and PHVB/Ho (*n* = 5), along with one control group, PHBV (*n* = 5), all of which underwent biopsies that included the entire thickness of the skin and the panniculus carnosus of the mid-dorsal area of the mouse. Cylindrical pieces of each membrane, measuring approximately 7 × 0.2 mm, were placed in the wound bed and covered with a transparent dressing. No topical treatment was administered during the control process, nor were the implants changed during the healing period. **Results**: Univariate and multivariate analyses were performed. The data show that the PHBV/Ho scaffolds reduce the diameter of the wounds by 100% after 40 days (*p* < 0.001), compared with PHBV/Av (100%; *p* = 0.211) and the control group, PHBV. **Conclusions**: From a macroscopic perspective, the PHBV/Ho scaffold significantly accelerated wound healing when applied once to the wound bed, outperforming both the PHBV/Av composite and PHBV alone. Notably, this effect was achieved without the need for dressing changes or additional treatment during the healing period.

## 1. Introduction

Several research studies have demonstrated the efficacy of bee honey and Aloe vera in the healing of different skin wounds due to their synergistic physicochemical properties, including antibacterial, anti-inflammatory, and antioxidant [1], and healing process stimulation traits [2,3,4,5,6,7]. In recent years, several empirical studies have demonstrated that both the single use of these natural products and their combinations with other treatments are effective in treating various types of partial-thickness and full-thickness wounds [4,8,9,10,11,12,13].

The technique for creating wounds that heal by secondary intention, similar to human skin, was described by Davidson (2013) [14] and Ren (2012) [15]. They inserted a subcutaneous silicone ring into the dorsal area of the mouse, creating a wound by performing a biopsy of the entire skin thickness, including the panniculus carnosus in the central region of the ring. They then sutured the upper edges of the wound to the inner edge, thereby preventing rapid closure due to the action of the panniculus carnosus, a layer of skeletal muscle in mice’s skin that assists wound healing through contraction. Without this layer, a secondary intention wound closure effect similar to that of human skin would be achieved.

In a mouse model treated in vivo with honey, the healing and closure rate was better on day 12 compared with a commercial wound dressing, AquacelAg (ConvaTec Inc., Reading, UK) [16]. Hernández-Rodríguez et al. (2023) [17] demonstrated a similar effect of using Aloe and pure honey in a mouse wound healing model without the panniculus carnosus over 50 days, mirroring the human wound healing process.

The high osmolality and low water (Aw) activity of honey, along with its low pH (3.2–4.5), contribute to these effects [18,19,20,21,22]. Additionally, the presence of organic acids (hexadecanoic, formic, propionic, gluconic, acetic, and benzoic acid) and phenolic and flavonoid compounds, as well as the combination of hydrogen peroxide and benzoic acid—which produces very stable peroxide compounds in the presence of endogenous catalase—creates a strong antibacterial effect [9,12,13,18,19,20,21,22,23,24,25,26,27,28,29,30,31,32] (Table 1). Bonsignore et al. (2024) emphasize that honey’s antibacterial, anti-inflammatory, antioxidant, and immunomodulatory properties arise from the combined action of its constituents, and that attempts to isolate or purify individual compounds may compromise its therapeutic potential [32]. This synergistic mechanism is particularly relevant in wound healing, where honey’s multifactorial effects—such as moisture retention, antimicrobial action, and stimulation of tissue regeneration—are most effective when the whole matrix is preserved [32].

In contrast, A. vera, a commercially processed product, has been described as a healing agent for superficial wounds [33,34]. It exhibits angiogenic and anti-inflammatory effects [8,35,36,37,38], which favor the proliferation and migration of keratinocytes and collagen deposition, triggering different healing phases and achieving sufficient epithelialization [7,35,36,37,38,39] (Table 1).

Poly (3-hydroxybutyrate-co-3-hydroxyvalerate) (PHBV) has been utilized since the 1960s in biomedical applications such as sutures and prosthetic devices due to its biocompatibility and biodegradability [40]. Ibrahim (2021) describes the chemical composition and molecular structure of PHBV, pointing out its potential applications in various fields, including packaging, tissue engineering, and drug delivery systems [41] (Table 1). Raza et al. (2020) [40], Ibrahim et al. (2021) [41], Das et al. (2019) [42], and Rodríguez-Cendal et al. (2023) [43] describe PHBV as a natural, biodegradable, and biocompatible copolymer belonging to the family of polyhydroxyalkanoates found in algae and certain bacteria, synthesized through bacterial fermentation, and consisting of two monomeric units: 3-hydroxybutyrate (3HB) and 3-hydroxyvalerate (3HV) (Table 1). It serves as an intracellular storage medium and has a hydrophobic surface along with a slow degradation rate. However, when combined with biomaterials, its mechanical and biophysical properties are enhanced, allowing for the synthesis of structures that serve as scaffolds for tissue regeneration or drug delivery [40,41,42,43].

One significant property of PHBV is the presence of microspheres, which impart unique characteristics, such as slow degradation through amorphous zones without the polymer breaking down [43]. This feature provides advantages for the release of products encapsulated in these microspheres. Chen et al. (2012) [44] demonstrated that PHBV microspheres serve as effective scaffolds for the regeneration of various types of neuronal cells in neuronal cultures, a result also observed in the experiments by Romero et al. (2024) [45,46].

The use of biocompatible natural and synthetic polymers as scaffolding materials has been extensively investigated for their ability to support cell migration and enhance the healing of full-thickness wounds by secondary intention. In addition, the integration of smart delivery systems capable of releasing natural products or therapeutic agents has further expanded their potential in regenerative medicine and wound management [41,42,43,47,48,49,50].

Natural and synthetic biomaterials have been widely employed to mimic the microarchitecture of the extracellular matrix (ECM), providing structural support and suitable scaffolds for neurite extension and tissue regeneration [40,51,52].

Electrospinning has emerged as a versatile technique for fabricating nanofibrous scaffolds from aqueous blends of polymers such as polyvinyl alcohol (PVA), chitosan, cellulose acetate, polycaprolactone (PCL), and silk, often combined with bioactive agents like honey to enhance wound healing [48,49,50,51,52]. This approach has also been extended to other regenerative applications [53,54,55,56]. More recently, hybrid electrospun scaffolds incorporating *Aloe vera* with polymers such as PCL, collagen, chitosan, and PVA have demonstrated potential as bioactive matrices for skin regeneration [45,46,53,56,57,58,59]. In parallel, the application of PHBV in tissue engineering has expanded significantly with the advent of electrospinning, enabling the design of fibrous architectures that mimic the native extracellular matrix and support cellular regeneration in wound healing contexts [43].

Electrospun nanofibers can be fabricated with either aligned or random orientations, effectively replicating the structural features of the ECM found in neural and other tissues [45,46,47,51]. By modulating the electrospinning parameters, fiber alignment and diameter can be precisely controlled, enabling the design of scaffolds that influence cellular behavior. Although the nanometer scale is commonly defined as up to 100 nm, broader definitions extend this range to 1000 nm when considering functional and biological effects [54,55]. In the context of neural tissue engineering, both aligned and non-aligned nanofibrous scaffolds have demonstrated the ability to guide neurite outgrowth, with aligned fibers significantly enhancing directional neurite extension, a critical factor for promoting functional neural regeneration [46,47,48,57].

The tissue response to PHBV polymeric implants in biopsies from scarless skin regeneration models was comparable to that observed with silk implants. Notably, the inflammatory reaction was less pronounced than that elicited by intramuscularly implanted PHBV-based scaffolds maintained for over one year. Furthermore, PHBV implants did not trigger an acute inflammatory response at the implantation site [40,41,43,47,48,49,50,51,52].

However, none of the studies consulted in this research have used a combination of hybrid scaffolds made of PHBV, honey, and A. vera for wound healing, thus creating a molecular structure that mimics the extracellular matrix as structural support for tissue regeneration. Our scaffolds function as implants inserted into the wound bed and do not require modification during the healing process. They are likely to slowly transport and release the natural compounds of honey or Aloe vera encapsulated in microspheres through diffusion into the amorphous regions of the material without degrading the matrix, allowing its structure to support the cells and maintain their adhesion, as pointed out by Rodríguez-Cendal et al. (2023) and Asl et al. (2021) [43,53].

Building upon these findings, we have developed and patented two distinct electrospun scaffolds incorporating Aloe vera or honey into poly(3-hydroxybutyrate-co-3-hydroxyvalerate) (PHBV) scaffolds, PHBV/Av and PHBV/Ho, respectively [60,61]. These hybrid systems aim to combine the structural and biodegradable properties of PHBV with the bioactive potential of natural additives, offering promising platforms for skin regeneration and neural tissue engineering.

This study aimed to macroscopically evaluate and compare the healing performance of three electrospun scaffolds—PHBV, PHBV/honey (PHBV/Ho), and PHBV/*Aloe vera* (PHBV/Av)—in full-thickness skin wounds over a 40-day period.

## 2. Materials and Methods

### 2.1. Animals

In accordance with the proposal to reduce the number of animals required without compromising statistical significance, according to the principles of the 3Rs (replacement, reduction, and refinement) [62] and by the Directive 2010/63/EU on the protection of animals used for scientific purposes [63], as well as previous studies that used murine models of similar wounds, which demonstrated significant differences with comparable group sizes [17,64], eight-week-old male CD15 Swiss mice (*n* = 15), averaging 41.2 g in weight, were randomly selected for experiments. The mice were housed in individual cages with unrestricted access to food and water dispensers. They were cared for in accordance with the European Directive previously mentioned. The animals were randomly divided into two experimental groups and one control group of five animals (*n* = 5) per group.

The Animal Ethics and Well-being Committee of the University of Las Palmas de Gran Canaria (ULPGC) approved the experimental procedures (Ref. 004/2013CEBA ULPGC).

Before and after the surgical procedure, the weight of the mice was measured. Glycemia was assessed using a standard glucometer (FreeStyle Optium Neo de Abbott^®^, Oxfordshire, UK) with a sample extracted from the lateral tail (coccygeal) vein. Body temperature was recorded with an infrared thermometer (TZL-801A, Shenzhen, China), and the diameter of the wound was gauged with a manual Vernier© caliper. These measurements were complemented by images captured from a tripod at the same distance using a smartphone (Samsung S5) camera (Samsung Electronics Co, Ltd., Suwon, Republic of Korea) while the animals were positioned on millimeter graph paper for scale. Observations were conducted at various time points ([t0]: immediately following surgery, [t7]: 7 days, [t15]: 15 days, [t20]: 20 days, and [t40]: 40 days) [17,36,65,66,67] by measuring the size of the wounds based on the average of two measurements taken perpendicular (d1) and parallel (d2) to the mouse’s backbone. Consequently, 20 observations were made per mouse, totaling 260 observations (Supplemental Data, Table S1).

Additionally, we assessed if the granulation tissue was pink and clean, if the wound bed showed total or partial slough or cellular debris, and if the wound exhibited complete closure covered by epithelial tissue with plentiful hair follicles.

Images were analyzed with the image analysis software package ImageJ^®^, version 2.16.0/1.54p [68].

### 2.2. Surgical Procedure

The animals were anesthetized intraperitoneally with 0.03 mL of medetomidine (1 mg/kg; 0.03 mg) and 0.06 mL of ketamine (100 mg/kg; 3 mg). For revival, 0.01 mg/kg of atipamezole was administered. Tramadol was given ad libitum (25 mg/kg) orally [69].

**Figure 1:**
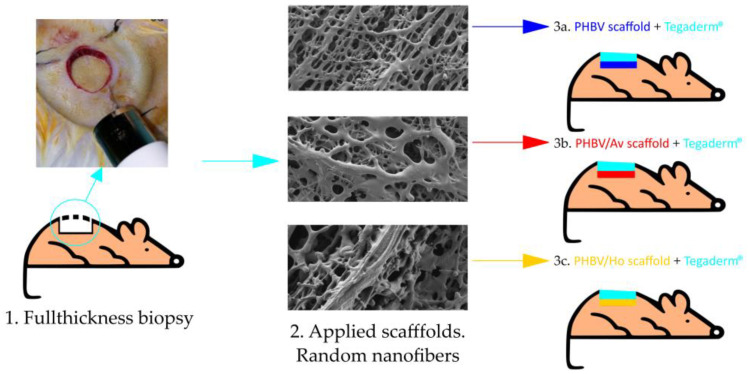
Protocol (Scale bar 5 mm).

**Figure 2:**
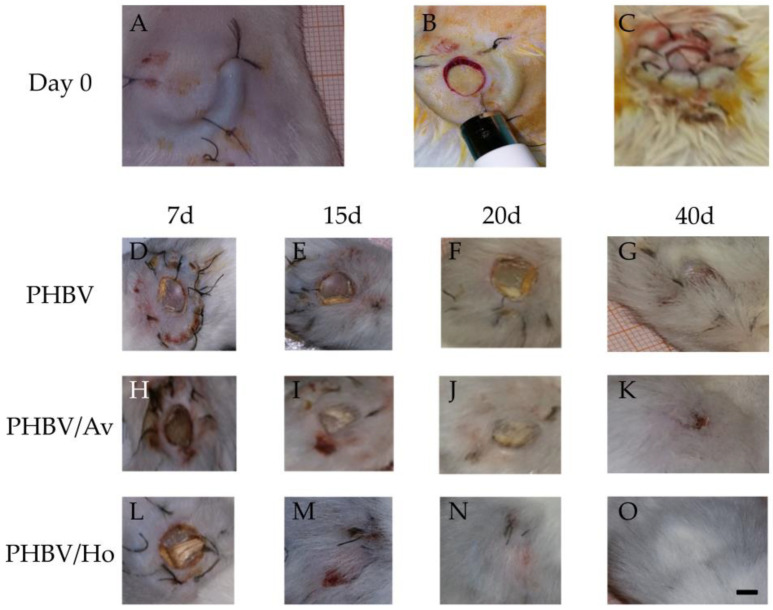
Anatomical views of the dorsal skin of mice during surgery and the wound healing process. (**A**–**C**) Surgical procedure: dorsal skin exposure and wound creation. The rostral side of the animals is oriented upwards in all images. (**B**) Placement of an 8 mm diameter subcutaneous nitrile ring prior to full-thickness skin excision using a Tru-Cut biopsy punch. (**C**) The nitrile ring was secured with additional sutures before initiating the topical scaffold treatment. (**D**–**G**) Wound healing progression in the PHBV group at days 7, 15, 20, and 40. Healing was slow and uneven, with a friable wound bed observed at days 15–20. The wound remained open at day 40 (**G**). (**H**–**K**) Wound healing in the PHBV/Av group showed a gradual reduction in wound size, though complete closure was not achieved by day 40 (K). (**L**–**O**) The PHBV/Ho group exhibited a more dynamic healing response. By day 15 (**M**), wounds contained granulation tissue, were nearly closed by day 20 (**N**), and fully healed with hair follicle coverage by day 40 (**O**). All animals were photographed on laminated millimeter grid paper for scale reference. Scale bar: 5 mm (applies to all images).

A small, sterile subcutaneous nitrile ring (10 mm in diameter, Figure 1 and Figure 2A) was inserted into the dorsal area of the animals and securely fixed subcutaneously in each animal to study the tissue repair mechanisms related to this type of wound and to regulate and control the contraction occurring in the skin of these animals during healing [14,15,17].

Ten days after insertion, a full-thickness wound was created on the skin within the ring, including the panniculus carnosus, using the same anesthetic procedure described above. A sterile skin biopsy punch (8 mm in diameter) was used (Ref. 94158BP-80-FA) (Figure 1 and Figure 2B). The internal edge of the ring was sutured to secure the wound and prevent skin contraction, following the experimental models of Davidson et al. (2013) [14], Ren et al. (2012) [15], and Hernández-Rodríguez et al. (2023) [17] (Figure 2C). Thus, the model simulates the biological processes of human wound healing.

### 2.3. Scaffold Fabrication and Morphological Characterization

Electrospinning is a well-established technique for producing polymeric nanofiber scaffolds. It involves applying a high-voltage electric field between a metallic needle and a grounded collector while a polymer solution is continuously delivered through a syringe. The electrostatic forces overcome surface tension, stretching the polymer solution into fine jets. As the solvent evaporates during flight, solid fibers are deposited onto the collector, forming a nonwoven, nanofibrous mat [70,71].

In this study, three types of non-aligned electrospun scaffolds were fabricated using poly(3-hydroxybutyrate-co-3-hydroxyvalerate) (PHBV; Sigma-Aldrich (St. Louis, MO, USA), Prod. No. 403121, CAS No. 80181-31-3). The control scaffold consisted of pure PHBV, while the other two were hybrid formulations incorporating either Aloe vera biogel (99% purity, organically cultivated; Aloe Park Tenerife (Arona, Spain), Luciano Reverón e Hijos S.L.) or natural multifloral honey (Cuevas de Guayadeque, Ingenio, Gran Canaria, Spain; Sanitary Registration No. E23.03229/GC/CEE), referred to as PHBV/Av and PHBV/Ho, respectively. Detailed fabrication parameters are described in the corresponding patents EP 3428117 [60] and EP 3461788 [61]. The resulting circular membranes measured 7 mm in diameter and 0.2 mm in thickness.

The surface morphology of the scaffolds was analyzed using field emission scanning electron microscopy (FESEM) with a Zeiss Sigma 300VP microscope (Carl ZEISS, Jena, Germany) at the SIMACE facility, University of Las Palmas de Gran Canaria. Samples were mounted on cylindrical aluminum stubs using double-sided carbon adhesive tape and imaged using a secondary electron detector at low accelerating voltages (0.8–1.5 kV). Representative images of each scaffold were acquired at various magnifications to assess fiber morphology and surface topography.

### 2.4. Control and Experimental Treatments

Each membrane piece, approximately 1 cm in diameter, was sterilized by irradiation with a commercial ultraviolet (UV) germicide lamp (λ = 254 nm) at a distance of 25 cm for 35 min [72]. The wounds in the first group (*n* = 5) were treated with saline (SF) or PHBV polymers only. This group served as the control.

The polymer scaffold PHBV/Av served as the second experimental group (*n* = 5), while the third experimental group (*n* = 5) was treated with the scaffold PHBV/Ho.

No topical treatments were applied, nor were any modifications made to the scaffolds during the control process. The wounds of the experimental groups were covered with a Tegaderm^®^ (St. Paul, MN, USA) transparent dressing (Figure 1). Observations were recorded at different time points ([t0]: immediately following surgery, [t7]: 7 days, [t15]: 15 days, [t20]: 20 days, and [t40]: 40 days), and measurements were taken for weight, temperature, glycemia, and wound size (Figure 2D–O).

The data were finally quantified using ImageJ^®^ software on the Fiji platform for biological image analysis [73].

### 2.5. Histological Analysis

Regenerated tissues were sectioned into four equal parts each, fixed in Zamboni’s fixative, and stored in an ultra-freezer at −40 °C. Selected tissue samples were processed for semithin sectioning and examined using optical microscopy.

For semithin section preparation, tissue samples were first rinsed in phosphate-buffered saline (PBS) and fixed overnight in 2.5% glutaraldehyde (Electron Microscopy Sciences (Hatfield, PA, USA), EM grade, 16220). Following two PBS washes, samples were post fixed in 2% osmium tetroxide (EMS, 19170) for 3 h. Dehydration was carried out through a graded ethanol series (20%, 40%, 60%, 70%, 96%, and 100%), followed by immersion in a 1:1 (*v*/*v*) mixture of absolute ethanol and propylene oxide (EMS, 20401), then pure propylene oxide, a 1:1 (*v*/*v*) mixture of propylene oxide and Embed812 epoxy resin (EMS, 14120), and finally pure epoxy resin overnight. Semithin sections (1 µm) were obtained using a Leica EM UC7 ultramicrotome. Sections were stained with Toluidine Blue (Panreac (Barcelona, Spain), 251176) and, alternatively, with a polychromatic method using Toluidine Blue–Basic Fuchsine (Sigma-Aldrich, 215597).

Semithin sections images were acquired using a ZEISS Axio A1 optical microscope (Carl ZEISS, Germany) at the SIMACE microscopy facility.

### 2.6. Statistical Analysis

Univariate analyses. The percentage variations in wound surfaces between the first and last days were summarized as medians and interquartile ranges (IQR = 25th–75th percentiles). Multiple comparisons among the treatments were conducted using nonparametric methods.

Multivariate analysis. A multivariate analysis was used to evaluate the effect of the treatments on the wound surface on each control day, which was ultimately adjusted for weight, temperature, and glycemia level. Since the design employed a repeated measure, the data were analyzed using mixed models. The wound surface was transformed logarithmically. Thus, we denoted *Wound* by “*mouse*, *treat*. *day*” and the wound surface for the mouse by “*mouse*” receiving the treatment “*treat*” at day “*day*.” According to Laird and Ware (1982) [74], we considered the following mixed model:

$$Log\;wound\;(mouse,treat.day)=\theta +\tau (treat)+\beta .day+\sum_{k}\gamma k.Zk+mouse+e\;(mouse,treat.day)$$

where *τ*_treat_ denotes the treatment effect (*τ*_reference_ = 0); *mouse* is the random effect of the mouse, which we assume is usually distributed with mean zero and standard deviation *σ* and (mouse,treat.day) is the error term. In the analysis, the covariables weight, temperature, and glycemia level were introduced. Next, variables were selected based on the Akaike Information Criterion (AIC) [75].

Therefore, we denoted the variables by _k_ that were finally maintained in the model after the selection. The model was estimated using the restricted maximum likelihood and summarized as coefficients, standard errors (SE), and *p*-values.

The use of repeated measures over five time points (t0, t7, t15, t20, and t40) and the application of mixed-effects models allowed us to increase statistical power by accounting for intra-subject variability.

Statistical significance was established at *p* < 0.05. Data were analyzed using the R package version 3.1.1 [76].

## 3. Results

### 3.1. Animals

The parameters of weight, temperature, and blood glucose levels were consistent across the three groups of animals. The final sample included 8-week-old animals (*n* = 13) with an average weight of 41.4 g (SD: 3.1). Two animals were excluded from the Aloe experimental group because they could not tolerate the inserted ring. Figure 1 shows the protocol followed with the animals, and Figure 2 illustrates the evolution of wound size in relation to the type of polymer applied and the number of control days. The PHBV/Ho experimental group demonstrated better efficacy in terms of healing time compared with the control group and the other experimental group (PHBV/Av).

Wound evolution was slow and highly irregular from day 7 in the control group (PHBV). A friable wound bed was observed on day 15, characterized by the absence of discharge and the formation of an incomplete skin surface with a rigid appearance (Figure 2D–G). Moderate hair follicles were noted in a partially healed area on day 40 (Figure 2G). The average sizes of the wounds for this group were 7.5 mm and 5.8 mm on days 7 (t7) and 15 (t15), respectively (Figure 2D,E), 4 mm on day 20 (t20) (Figure 2F), and 2.4 mm on day 40 (t40) (Figure 2G). The average weight was 38.6 g, glycemia was 122.24 mg/dL, and temperature was 34.5 °C.

The size of the wound in the PHBV/Av experimental group gradually decreased (t7 = 7.2 mm; t15 = 5.9 mm; t20 = 5 mm; t40 = 1 mm), showing better results than the control group, although the wound had not fully healed by day 40 (Figure 2K). The wound bed exhibited an incomplete skin surface with a rigid appearance (Figure 2H–K). The average weight of the animals was 44.8 g, the average temperature was 34.5 °C, and the average blood glucose level was 126 mg/dL.

We observed a gradual reduction in the initial average diameter of the scars during the progression of the wounds treated with PHBV/Ho (Figure 2L–O), beginning on day 7 (t7 = 7.9 mm) (Figure 2L) and continuing steadily through days 15 (t15 = 5 mm) and 20 (t20 = 2.4 mm) (Figure 2M,N). From day 15 to day 20, there was no secretion, and the wound bed remained clean and free of exudate (Figure 2L–N), with abundant hair follicles visible in the healed areas (Figure 2O). The average weight was 41 g, the average temperature was 34.1 °C, and the blood glucose level was 137 mg/dL.

### 3.2. Scaffold Fabrication and Morphological Characterization

**Figure 3:**
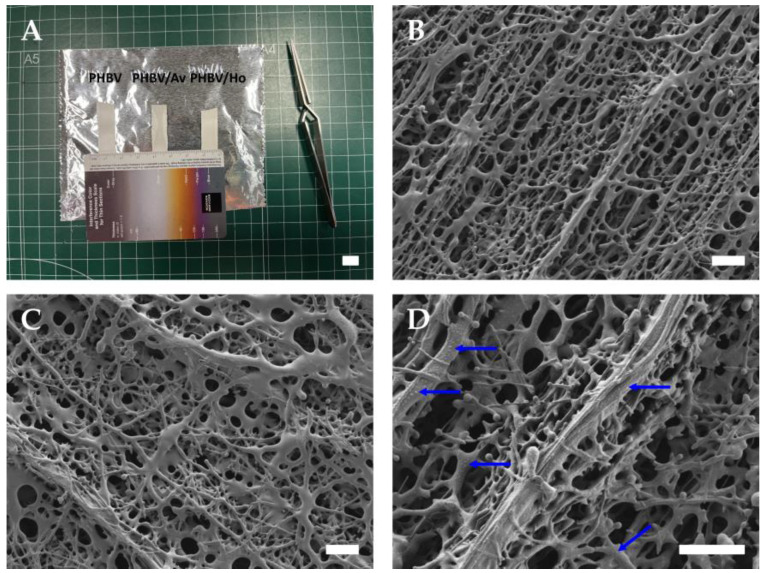
Ultrastructure of non-aligned PHBV nanofibers. (**A**) Macroscopic view of **PHBV** scaffolds. (**B**) Field emission scanning electron microscopy (FESEM) image of **PHBV** nanofibers. (**C**) FESEM image of **PHBV/Av** nanofibers. (**D**) FESEM image of **PHBV/Ho** nanofibers. **Scale bars**: 1 cm in panel (**A**), and 20 µm in panels (**B**–**D**).

Representative macroscopic images of the electrospun scaffolds are shown in Figure 3A. The surface morphology observed by field emission scanning electron microscopy (FESEM) is presented in Figure 3B–D, corresponding to PHBV (control), PHBV/Aloe vera (PHBV/Av), and PHBV/honey (PHBV/Ho), respectively. All scaffolds exhibited a disordered, interconnected nanofibrous network resembling a three-dimensional porous matrix. Both PHBV (Figure 3B) and PHBV/Av (Figure 3C) displayed smooth, homogeneous fibers with characteristic bead-like structures. The incorporation of Aloe vera did not significantly alter the fiber morphology compared with the control. Interestingly, this contrasts with previous reports on PVA/Aloe vera/chitosan blends, where Aloe vera induced notable topographical changes [76]. In contrast, the addition of honey markedly affected the scaffold architecture. PHBV/Ho (Figure 3D) exhibited an irregular surface with distinct ‘nano-bubble’ formations surrounding the fibers (indicated by blue arrows), suggesting a significant morphological impact of honey incorporation.

### 3.3. Histology

**Figure 4:**
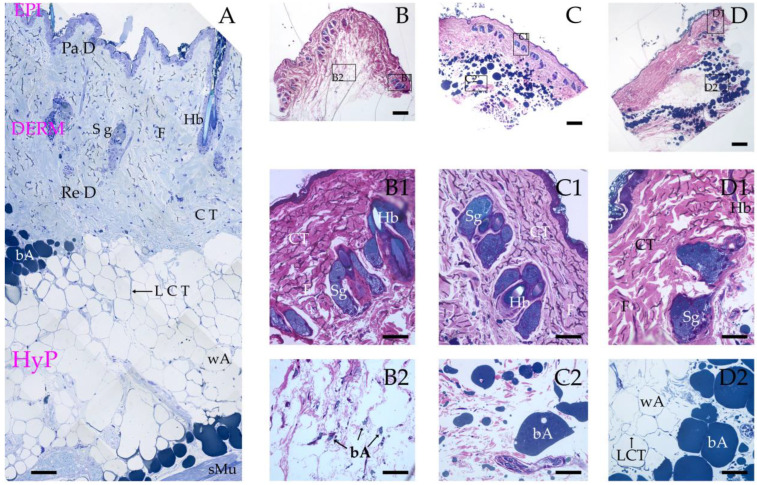
Semithin sections of skin biopsies. (**A**) Control biopsy. Panoramic view. (**B**) Biopsy treated with PHBV scaffold. Panoramic view. (**B1**) Detail of panel B showing the epidermis and dermis. (**B2**) Detail of panel B showing the hypodermis. (**C**) Biopsy treated with PHBV/Av scaffold. Panoramic view. (**C1**) Detail of panel (**C**) showing the epidermis and dermis. (**C2**) Detail of panel (**C**) showing the hypodermis. (**D**) Biopsy treated with PHBV/Ho scaffold. Panoramic view. (**D1**) Detail of panel (**D**) showing the epidermis and dermis. (**D2**) Detail of panel (**D**) showing the hypodermis. Scale bars: 50 µm in panels (**A**,**B1**,**B2**,**C1**,**C2**,**D1**,**D2**); 200 µm in panels (**B**,**C**,**D**).

A panoramic view of a semithin section of control skin (untreated) is shown in Figure 4A. Representative images of the skin biopsies containing the different scaffolds are shown in Figure 4B–D: PHBV (Figure 4B), PHBV/Av (Figure 4C), and PHBV/Ho (Figure 4D).

Figure 4A illustrates the three principal layers of healthy skin in a semithin section: the epidermis (EPI), dermis (DERM), and hypodermis (HyP). The dermis displays its characteristic papillary (PaD) and reticular (ReD) layers, where connective tissue (CT), fibroblasts (F), sebaceous glands (Sg), and hair bulbs (Hb) can be identified. In the hypodermis, the presence of loose connective tissue (LCT), white adipose tissue (wA), brown adipose tissue (bA), and striated muscle (sMu) is evident.

Light microscopy did not reveal the presence of polymeric material within the biopsies. Additionally, no foreign bodies were detected in any of the processed specimens, suggesting either the absence of rejection or complete degradation of the scaffold material by the host tissue. A plausible hypothesis is that the polymer underwent full bio resorption over the 40-day experimental period, leaving no detectable trace. This assumption warrants further confirmation by transmission electron microscopy (TEM) for detailed ultrastructural analysis.

The biopsies containing the different scaffolds (PHBV, PHBV/Av, and PHBV/Ho) show a reconstructed skin architecture, with all three layers—epidermis, dermis, and hypodermis—clearly distinguishable. The epidermis appears intact and free of morphological abnormalities in Figure 4(B1–D1). In the dermis, the presence of connective tissue, fibroblasts, hair bulbs, and sebaceous glands can be observed.

In contrast, the hypodermal layer (Figure 4(B2–D2)) exhibits notable differences when compared with the control. In PHBV-treated samples (Figure 4B2), the hypodermal structure is poorly defined, with an underdeveloped adipose layer characterized by sparse and immature adipocytes. The PHBV/Av group (Figure 4C2) shows a highly irregular hypodermis, containing scattered connective tissue and patches of gray adipose tissue, with no white adipose tissue present. In contrast, the PHBV/Ho-treated samples (Figure 4D2) exhibit a hypodermal morphology similar to that of healthy skin, with discernible white and gray adipose tissue, along with the typical loose connective tissue associated with this layer.

### 3.4. Statistical Analysis

Table 2 shows the variation in the wound surfaces from the first to the last day. This percentage reduction was complete in the PHBV/Ho group. Table 3 shows that the PHBV/Ho polymer treatment significantly differed from the PHBV polymer treatment alone (*p* < 0.001). The difference between the honey and *A. vera* treatments was quasi-significant (*p* = 0.0628). The glycemia levels and temperature were not statistically significant and, therefore, were not included in the model.

**Figure 5:**
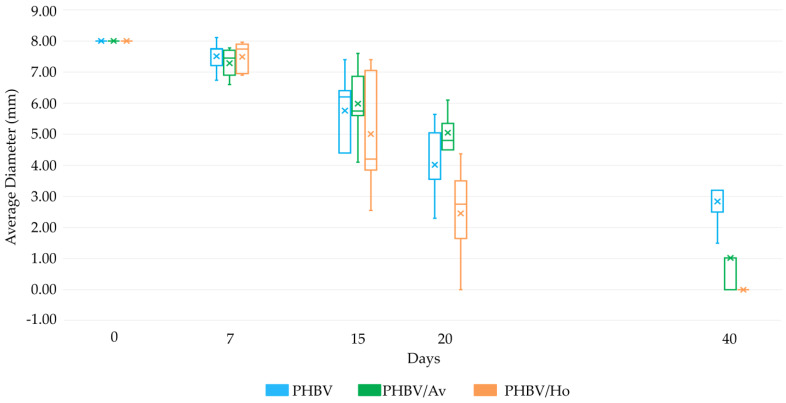
Evolution of wound size according to polymer treatment and duration of therapy.

Figure 5 indicates that the most significant reduction in wound surface evolution was associated with the treatment using PHBV/Ho.

Figure 5 shows a box plot representation of wound diameter (mm) over a 40-day treatment period with three different scaffolds: PHBV (blue), PHBV/Av (green), and PHBV/Ho (orange). The measurements were taken at days 0, 7, 15, 20, and 40. Each box plot displays the interquartile range (IQR), with whiskers indicating variability outside the upper and lower quartiles. The mean values are marked with an “×”. The PHBV/Ho group exhibited the most significant and consistent reduction in wound size, followed by PHBV/Av. The PHBV group showed slower and more variable healing progression. PHBV exhibited a gradual reduction in wound diameter over time, though healing remained incomplete by day 40. A moderate improvement in wound healing is achieved with PHBV. PHBV/Av demonstrated a more pronounced decrease in wound size, with lower median and mean values compared with PHBV at each time point. It has an intermediate effect between the PHBV and PHBV/Ho treatments. PHBV/Ho showed the most significant reduction in wound diameter, with minimal variability and near-complete closure by day 40. It accelerated the reduction in wound size.

These results suggest that the incorporation of *Aloe vera* and *honey* into PHBV scaffolds enhances wound healing efficacy, with PHBV/Ho showing the most consistent and accelerated tissue regeneration.

## 4. Discussion

The insertion of the subcutaneous ring in the dorsal area of the mouse’s skin allowed for the long-term monitoring of the evolution of the wound in the study of the healing process by secondary intention similar to that which occurs in human skin (Davidson et al., 2013 and Ren et al., 2012) [14,15], and is therefore shown to be an appropriate method for in vivo clinical monitoring of this process in murine models.

This study presents evolution data extending beyond the standard 7 to 15 days [14,48,52,74] to a span of 40 days [17] for excisional wounds in mice. The wound healing in the control group differed significantly from that in the two experimental groups. Overall, the quality of scar tissue in the honey group on day 40 was superior to that in the Aloe vera group. In contrast, the quality was lower in the control group.

In this regard, Rubiano-Navarrete et al. (2024) [57], Andreu et al. (2015) [77], Maleki et al. (2013) [78], and Abrigo et al. (2014) [79] highlight the utility of electrospun nanofibers as effective structures for promoting chronic wound healing. Specifically, Rodríguez-Cendal et al. (2023) [43] reviewed the biomedical applications of PHBV, emphasizing its role in drug encapsulation and sustained release systems, including applications for anticancer and anti-inflammatory agents. They demonstrate the use of PHBV nanocarriers for prolonged drug release, such as insulin delivery systems that maintain therapeutic levels for up to 27 days. This occurs due to the diffusion of the encapsulated compound through the amorphous regions of the spheres without the degradation of the matrix, thus preserving its original structure and granting the polymer the ability to facilitate cell adhesion and growth. 

Furthermore, several authors have emphasized the effectiveness of using these scaffolds alongside various products to achieve complete healing in full-thickness wounds [45,46,48,49,50,55,57,58,77,80,81,82].

Pilehvar-Soltanahmadi et al. (2018) indicate that incorporating natural substances into nanofibers through electrospinning techniques creates fibers that enhance the healing process by both providing a substrate for cellular support and enabling the in situ release of these products into the ulcerous niche [83]. This approach yields better results than using only the polymer without these added natural components, as their various properties directly influence the healing process by stimulating angiogenesis, promoting fibroblast production (effects of honey), or facilitating collagen remodeling (effects of Aloe vera) [83].

Similarly, the potential to synthesize the polymer in a reticular or aligned manner ensures dual functionality, enabling granulation tissue regeneration with the non-aligned form and promoting neuritic regrowth with the aligned form. Prabhakaran M. et al. (2013) [48] and Masaeli et al. (2013) [56] demonstrate the effectiveness of electrospun aligned PHBV/collagen nanofibers as substrates for nerve tissue engineering, indicating that these aligned fibers allow neurite regrowth.

Masaeli et al. point out the promising possibilities of using aligned PHBV nanofiber composites for nerve regeneration if compounds that stimulate this growth are added [56].

In this regard, Romero-Alemán et al. (2019, 2025) found that the use of aligned and non-aligned PHBV matrices, combined with Aloe vera and honey, promotes neurite regrowth as well as the regeneration of mouse skin after wounding [45,46].

Furthermore, the polymer’s characteristics aid in managing the water produced by honey’s osmotic action, even distributing it across the membrane’s surface. This process ensures that an optimal moisture level is maintained for effective healing and cell adhesion [40,41,42,43,44,46,48,49,53,56,57,58].

In our study, we observed that wounds treated with nanofibers incorporating the natural compounds Aloe vera and honey produced better results than those treated with the polymer alone. Additionally, we noted that wounds treated with the PHBV polymer and honey healed more quickly than those treated with the PHBV polymer and Aloe. In a study by Hernández-Rodríguez et al. (2023), it was found that using these natural products in their pure form for wound healing yielded different outcomes, with better healing dynamics noted with Aloe than with honey, compared with traditional hydrocolloid dressings [17]. However, this study found that when these natural products were combined with the PHBV polymer, the healing dynamics were significantly more favorable with the polymer combined with honey than with aloe or the polymer alone [40,41,43].

On this matter, Raza et al. (2020) [40] and Rodríguez-Cendal et al. (2023) [43] point out that PHBV is a hydrophobic natural polymer that, when combined with biomaterials, enhances their mechanical and biophysical properties. This combination allows, among other actions, a slow and gradual release of the products encapsulated in the microspheres without degrading the polymer itself. We hypothesize that this property would enable the release and synergistic action of the compounds in honey, facilitating progressive activation through their natural antimicrobial properties (peroxidase activity with H_2_O_2_ production, osmotic effects due to high sugar concentration, low water activity, high acidity (pH 3–4), and presence of the defensin-1 peptide), proteolytic enzyme activity, and anti-inflammatory actions due to the presence of polyphenols and flavonoids (more abundant in dark honeys) that exert antioxidant effects, etc. [32,59]. All these effects, which activate consecutively and synergistically without one standing out above the others, combined with the hydrophobic property of the polymer described above, enhance the polymer’s ability to absorb excess moisture and maintain a suitable humidity environment that prevents the wound edges from macerating, thus allowing the stem cells at the edges to continue the skin repair process at different stages faster than with the other polymers used [17,40,41,43,47,57,58,59].

This scaffold combination of PHVB and honey fosters quicker wound healing under improved conditions compared with the one made from Aloe vera or the control, as illustrated in Figure 4.

Based on the results obtained and the previous literature, we conjecture that the PHBV/honey scaffold primarily enhances antimicrobial, fibrinolytic, and anti-inflammatory mechanisms during the initial stages of healing. The rapid cleansing of the wound bed, the absence of exudate from day 15 onwards, and the formation of active granulation tissue suggest a synergistic action of the bioactive compounds present in honey, such as hydrogen peroxide, organic acids, phenolic compounds, and autolytic enzymes [9,11,12,13,19,24,28,30,32,40,41,43]. These components not only inhibit bacterial growth but also promote the elimination of cellular debris and modulate the inflammatory response, creating an environment conducive to tissue regeneration. Although antioxidant and angiogenic effects are also recognized [84], we believe that antimicrobial action and inflammation regulation are the processes most enhanced by the proposed system.

It is important to note that human skin takes approximately 60 to 90 days to heal, depending on various factors [85,86]. Therefore, utilizing this combination of PHBV polymer and honey may represent an intriguing strategy to explore for enhancing the healing of secondary intention wounds and chronic wounds [57,78,79,83,86,87].

Furthermore, glycemia levels, temperature, and weight parameters were within physiologically normal limits [88].

Forty days after lesioning, scaffolds containing honey and A. vera accelerated wound closure. The most significant differences in wound diameter among the groups were observed at 20 and 40 days post lesion (Figure 2D–O). Consequently, the PHBV/Ho group exhibited enhanced dynamic wound healing (Figure 2L–O).

The compounds of the PHBV/Ho and PHVB/Av nanofibers will be released in a controlled manner, promoting cell growth. Scaffolds with honey contributed to faster healing than the others, as observed in the image sequence and graphics (Figure 2A–O; Figure 5). As other authors have described, this may be linked to the antibacterial and granulation tissue promotion properties of honey [9,12,28,29,31,32,59]. The enzymatic and autolytic properties of honey [13,21,26,32,59] eliminate cellular debris by activating plasmin and proteases, enabling the digestion of fibrin layers adhering to the wound bed and facilitating the healing process, as demonstrated in a study by Hernández-Rodríguez et al., (2023) [17]. This process is supported by the web-like arrangement of the fibers, leading to the development of active granulation tissue, which results from previous angiogenesis, fibroblast migration, and collagen deposition [32,52,53,59].

The scaffold PHBV is a polyester derived from polyhydroxyalkanoate produced by microorganisms under unbalanced growth conditions [41,42,52]. It possesses desirable properties, including a high surface-area-to-volume ratio, adequate mechanical stability, and sufficient pore size in the resulting nanofibrous scaffolds. The high porosity of this nanofiber enables oxygen and water permeability, as well as nutrient exchange. It effectively removes metabolic waste and prevents fluid accumulation at the wound site since nanofibrous dressings absorb wound exudates much more efficiently than film-type dressings. Hence, the porous structure allows the appropriate permeation of atmospheric oxygen into the wound [40,41,42,43,52,79,82,84]. Additionally, high surface areas foster the attachment of fibroblasts and endothelial cells, along with their subsequent proliferation and differentiation during tissue regeneration [43,50,52,53,59,77,79,84]. Contact with blood activates the coagulation and complement systems, which is essential as it stimulates the initial phase of wound healing [87]. In our study, PHBV/Av and PHBV/Ho scaffolds seem to provide a favorable scaffold for cell proliferation, as suggested by the previously cited authors.

On the other hand, the authors of [40,41,42,43,52,79,84] demonstrated that this type of PHBV nanofiber provides an excellent structure for the attachment and growth of chondrocytes as a cell culture surface for tissue engineering. This material did not cause any acute vascular reactions or adverse events at the implantation site, such as suppurative inflammation or necrosis, as shown in Figure 2. Its degradation into oligomers and monomers is not toxic to cells [47]. The degradation of these fibers likely occurs after the microcapsules release various compounds over an extended period [40,41,42,43,52,77,81,83,84,89]. In our case, the microcapsules may have released different compounds from honey and A. vera, facilitating a faster wound healing process with the honey-compounded polymer than with the Aloe vera-compounded polymer or the polymer without any natural product.

The PHBV/Ho group showed faster dynamic healing compared with the PHBV/Av group, with a gradual reduction in wound size (Figure 2L–O) until complete healing at 40 days. However, healing in the PHBV/Av group remained incomplete at 40 days (Figure 2K). In the PHBV group (control group), the degree of wound healing showed uneven and prolonged progression, resulting in a friable ulcerative bed at 15–20 days. The wound in this group remained open 40 days after injury (Figure 2G). This is illustrated in the histological images in Figure 4, which show healthy skin with a more organized basal structure in the PHBV/Ho biopsy samples (Figure 4D2). In contrast, the other biopsies reveal a less organized subcutaneous structure compared with that of the honey polymer (Figure 4B2,C2).

The most important achievement of this experiment was that the scaffold was placed only once, and no other significant treatment was performed on the original scar, which was sufficient for wound healing. Furthermore, without replacing the scaffold, after 40 days (approximately 5 weeks), this hybrid honey scaffold achieved complete wound healing by maintaining an adequately humid environment necessary for the healing processes, according to Pilehvar-Soltanahmadi et al. (2018) [83].

Our patented scaffolds, PHBV/Av and PHBV/Ho, infused with natural products, can maintain wound moisture and thereby support the contraction of autologous skin. Additionally, fluid and cell infiltration promote structural degradation and scarless remodeling (see Figure 4B1,C1,D1), present study) [60,61].

Therefore, they can have biomedical applications such as designing graft materials, coating endoprostheses and surgical meshes to enhance cell growth and adhesion, reinforcing sutures in poorly vascularized cartilaginous tissues, and serving as implants in ulcerated tissue due to dependency, including dental implants or coatings, wound dressings, and absorbable sutures, as suggested by different authors [45,46,50,59,77,78,79,82,83,85,89,90].

This is the first preclinical validation of the formulations used for manufacturing PHBV scaffolds with honey and PHBV with Aloe vera, applied in a murine secondary wound model that simulates human healing. It confirms their validity for this type of experiment, providing quantitative and qualitative evidence from macroscopic, histological, and statistical perspectives. The results show that the PHBV/Ho and PHBV/Aloe vera matrix accelerates healing compared with the control group (PHBV). For this reason, it was decided to protect this data with the cited patents. These experimental results represent an original scientific contribution that validates and expands the biomedical potential of the patent-protected products, which describe the composition and manufacturing method of the PHBV/Av and PHBV/honey hybrid scaffolds.

### 4.1. Antimicrobial Considerations in Wound Healing

Infection control is a critical factor in the healing of full-thickness wounds, particularly those healing by secondary intention. The presence of microbial contamination can delay epithelialization, increase inflammation, and lead to chronic wound states. In this context, the antimicrobial properties of both honey and Aloe vera are of particular relevance. Honey exhibits broad-spectrum antibacterial activity due to its low pH, high osmolarity, hydrogen peroxide content, and the presence of phenolic compounds and flavonoids. These components act synergistically to inhibit the growth of both Gram-positive and Gram-negative bacteria, including antibiotic-resistant strains such as MRSA. Aloe vera also contributes to antimicrobial defense through compounds such as anthraquinones, saponins, and acemannan, which have demonstrated bacteriostatic and fungistatic effects [27,28,30,31,32,36,37,43].

Although microbiological cultures were not performed in this study, the absence of exudate, odor, or signs of infection in the PHBV/Ho and PHBV/Av groups throughout the 40-day period suggests that the antimicrobial properties of these natural compounds contributed to a favorable healing environment. These findings are consistent with previous reports highlighting the role of honey and Aloe vera in reducing microbial load and promoting tissue regeneration.

### 4.2. Clinical Perspective and Translational Outlook

The findings of this study may have significant implications for the treatment of chronic and complex wounds, particularly those healing by secondary intention, such as pressure ulcers, diabetic foot ulcers, venous leg ulcers, and post-surgical wounds with tissue loss. These wound types often present challenges related to infection control, delayed epithelialization, and poor vascularization—factors that the bioactive properties of honey and Aloe vera may help address when delivered through a biodegradable scaffold.

To advance toward clinical application, several preclinical steps are necessary. These include scaling up scaffold production under Good Manufacturing Practice (GMP) [91] conditions, conducting biocompatibility and toxicity studies in larger animal models, and performing molecular analyses to confirm the activation of key regenerative pathways. Additionally, the reproducibility and stability of scaffold composition must be validated across batches. Ultimately, early-phase clinical trials will be needed to assess safety, efficacy, and patient outcomes in a real-world setting.

### 4.3. Limitations

This study acknowledges several limitations. First, the sample size was relatively small (*n* = 5 per group), which, although aligned with ethical guidelines for animal research and supported by statistical modeling, may limit the generalizability of the findings. Second, the analysis focused primarily on the macroscopic and histological outcomes; the molecular markers of inflammation, angiogenesis, or tissue remodeling were not assessed. Including such markers in future studies would provide deeper insights into the underlying biological mechanisms. Lastly, while the experimental design was carefully controlled, the reproducibility of the results in other models or clinical settings remains to be validated. Further studies with larger cohorts, molecular profiling, and long-term follow-up are warranted to confirm and expand upon these findings.

## 5. Conclusions

The PHBV/honey (PHBV/Ho) scaffold demonstrated superior efficacy in promoting wound closure compared with both the control PHBV and the PHBV/*Aloe vera* (PHBV/Av) scaffolds. From a macroscopic perspective, PHBV/Ho significantly accelerated the healing of full-thickness skin wounds following a single application, without the need for dressing changes or additional interventions throughout the 40-day period. Both hybrid scaffolds supported cell migration and enhanced tissue regeneration, contributing to scar-free healing and the recovery of skin appendages. Moreover, the preliminary observations suggest a potential role in facilitating axonal regrowth, highlighting their promise for broader applications in regenerative medicine. These findings support the potential of PHBV-based hybrid scaffolds, particularly those incorporating honey, as effective and practical biomaterials for wound healing therapies.

### Future Perspectives

The promising results obtained with the PHBV/Ho scaffold highlight the potential of natural compound-based hybrid biomaterials in regenerative medicine. Future studies should focus on elucidating the molecular mechanisms underlying the enhanced healing response, particularly the role of honey-derived bioactive compounds in modulating inflammation, angiogenesis, and tissue remodeling. Additionally, long-term in vivo studies are warranted to assess the biocompatibility, biodegradation kinetics, and functional integration of these scaffolds in more complex wound models. Expanding the application of these materials to neural tissue repair, guided axonal regeneration, and chronic wound management could further validate their clinical relevance. Finally, scaling up the fabrication process and evaluating the scaffolds under the Good Manufacturing Practice (GMP) conditions will be essential steps toward clinical translation.

## 6. Patents

Due to this research, the following patents were applied for and obtained:

1. Monzón-Monzón, M., Romero-Alemán, M.M., Hernández_Rodríguez, JE., Pérez-Galván, JM. Hybrid Aloe vera nanofibers. EUROPEAN PATENT OFFICE. EP 3461788;12.10:2022. Munich.

2. Monzón_Monzón, M., Romero-Alemán, MM., Hernández-Rodríguez, JE., Pérez-Galván, JM. Hybrid honey nanofibers. EUROPEAN PATENT OFFICE. EP 3428117;27.07:2022. Munich.

## Figures and Tables

**Table 1:** Main components of honey and Aloe vera, the chemical structure of PHBV, and how the ratio of 3HB to 3HV affects the polymer’s flexibility and degradation rate. 3HB: 3-hydroxybutyrate; 3HV: 3-hydroxyvalerate.

	Main Components	
Honey	Aloe Vera	PHBV
A complex natural substance composed mainly of:	A complex natural substance composed mainly of:	It is a random copolymer of 3HB and 3HV
Sugar (fructose 38%; glucose 31%)	Polysaccharides (e.g., acemannan)	monomer units:
Water (17%)	Glycoproteins	3-hydroxybutyrate; CH_3_-CH(OH)-CH_2_-COO^−^
Organics acids (e.g., gluconic acid)		3-hydroxyvalerate; CH_3_-CH_2_-CH(OH)CH_2_-COO^−^
Enzymes (e.g., glucose oxidase, catalase)		
Phenolic compounds and flavonoids		
Vitamins and minerals		
Defensin 1 peptide		

**Table 2:** Variation in wound surfaces from the first day to the last day.

Treatment (No.)	Day 0 (First)	Day 40 (Last)	Percent Reduction
PHBV	8 (8, 8)	2.5 (2.5, 3.2)	68.8 (60, 68.8) ^a^
PHBV + Honey	8 (8, 8)	0 (0, 0)	100 (100, 100) ^b^
PHBV + Aloe Vera	8 (8, 8)	0 (0, 1)	100 (87.2, 100) ^a,b^

Data are presented as medians (interquartile range [IQR]); different superscripts indicate significant differences at *p* < 0.05.

**Table 3:** A mixed model for the surface of deep injuries (logarithm scale) using PHBV as a control.

	Coefficient (SE)	*p*-Value
(Intercept) Treatment	2.334 (0.085)	<0.001
PHBV (reference)	0	-
PHBV + Honey	−0.382 (0.107)	<0.001
PHBV + Aloe Vera	−0.136 (0.109)	0.211
Time, per day	−0.054 (0.004)	<0.001

## Data Availability

The data for this research can be found in Table S1, Supplementary data.

## Supplementary Materials

The following supporting information can be downloaded at: https://www.mdpi.com/article/10.3390/pharmaceutics17070833/s1, Table S1: Data from observations collected for measured parameters. ID (identification); treatment (type of polymer applied); time (days); weight (grams); glycemia (mg/dL); temperature (temp °C), and wound size (mm).

## Abbreviations

The following abbreviations are used in this manuscript:

PHVB	Poly (3-hydroxybutyrate-co-3-hydroxy valerate)
PHBV/AV	Poly (3-hydroxybutyrate-co-3-hydroxy valerate)/Aloe Vera
PHBV/Ho	Poly (3-hydroxybutyrate-co-3-hydroxy valerate)/Honey
PBS	Phosphate-Buffered Saline
ECM	Extracellular matrix
PVA	Polyvinyl alcohol
PCL	Polycaprolactone
ULPGC	Universidad de Las Palmas de Gran Canaria
FESEM	Field Emission Scanning Electron Microscope
UV	Ultraviolet
AIC	Akaike Information Criterion
SE	Standard Errors
SIMACE	Advanced Confocal and Electron Microscopy Research Facility
