# Supplementary material for: Macroscopic Evaluation of Poly(3-hydroxybutyrate-co-3-hydroxy valerate), PHBV-Based Nanofiber Scaffolds with Aloe Vera or Honey in Murine Wound Healing
Source: Pharmaceutics. 2025 Jun 26;17(7):833. doi: 10.3390/pharmaceutics17070833 (PMC12299421; doi:10.3390/pharmaceutics17070833)
Supplement: Supplementary file 1 [file pharmaceutics-17-00833-s001.zip › pharmaceutics-3699928-supplementary.pdf]

Supplementary Materials

Table S1: Data from observations collected for measured parameters. ID. Identification; Treatment (type of polymer applied); Time (days); Weight (grams); Glycemia (mg/dL); Temperature (Temp ºC) and Wound size (mm).

ID	*Treatment*	*Time (days)*	*Weight gr)*	*Glycemia (mg/dL)*	*Temp (C °)*	*Injury (mm)*
	PHBV					
1	1	0	39.2	124	35.1	8
1	1	1	40	132	35.2	7.21
1	1	2	39.3	142	33.5	7.4
1	1	3	40	126	33.1	3.55
1	1	4	40.7	116	34.8	2.5
2	1	0	30.1	120	35.5	8
2	1	1	30.7	115	34.7	7.74
2	1	2	30.6	125	35	6.2
2	1	3	30	120	35	5.05
2	1	4	30.3	88	33.5	4.5
3	1	0	41.5	130	34	8
3	1	1	40.7	154	34.1	7.75
3	1	2	39.6	151	35.3	6.4
3	1	3	39.6	132	34.7	2.3
3	1	4	40.5	120	34.3	1.5
4	1	0	47.9	106	34.7	8
4	1	1	47.6	122	33.6	6.74
4	1	2	44.5	134	34.9	4.4
4	1	3	48.5	122	34.5	3.55
4	1	4	53.1	112	33.7	2.5
5	1	0	34.3	97	35.8	8
5	1	1	36.1	116	34.7	8.11
5	1	2	34.4	124	34.5	4.4
5	1	3	34	128	35	5.64
5	1	4	34	100	33.8	3.2
	PHBV MIEL					
1	2	0	43.7	117	32.9	8
1	2	1	43.6	143	34.6	7.96
1	2	2	43	164	34.6	7.4
1	2	3	42	138	34	4.37
1	2	4	43.3	127	34	0
2	2	0	40	139	31.9	8
2	2	1	41.5	138	34.8	7.74
2	2	2	39.4	144	34.3	4.2
2	2	3	39.6	139	34.4	3.5
2	2	4	41.4	113	34.2	0

3	2	0	42.7	136	33	8
3	2	1	43.3	136	34.4	6.95
3	2	2	41.4	145	34.6	7.05
3	2	3	42.7	164	34.2	1.65
3	2	4	43.9	179	34	0
4	2	0	36.9	133	32.6	8
4	2	1	38.2	128	35	6.9
4	2	2	34.8	126	35.1	3.85
4	2	3	35.8	151	35	2.75
4	2	4	37.6	140	34.2	0
5	2	0	38.9	137	32.8	8
5	2	1	40	131	34.7	10.15
5	2	2	38.9	128	35	2.55
5	2	3	38.1	122	34.9	0
5	2	4	39.1	117	35	0
	PHBV ALOE					
1	3	0	55.9	126	33.7	8
1	3	1	59.5	161	33.8	7.45
1	3	2	59.4	160	34.7	5.6
1	3	3	59	119	35.2	5.1
1	3	4	60.7	139	34.2	0
2	3	0	45.4	130	33,6	8
2	3	1	49	138	34	7.7
2	3	2	47.7	121	34.7	5.75
2	3	3	47.1	113	34	4.5
2	3	4	46.8	129	34.6	0
3	3	0	43	115	34.1	8
3	3	1	46.6	99	35.7	6.6
3	3	2	43.5	128	35	7.6
3	3	3	42.8	110	34.6	6.1
3	3	4	42.8	108	35	4.1
4	3	0	37.1	124	34	8
4	3	1	37.7	144	34.4	7.78
4	3	2	37	145	35.5	4.1
4	3	3	999	999	999	999
4	3	4	999	999	999	999
5	3	0	33.9	101	34.5	8
5	3	1	33.9	129	34.5	6.9
5	3	2	33.8	126	34.2	6.86
5	3	3	34.6	104	35	4.5
5	3	4	34.2	126	34.7	0
